# Use of medical therapies before pulmonary endarterectomy in chronic thromboembolic pulmonary hypertension patients with severe hemodynamic impairment

**DOI:** 10.1371/journal.pone.0233063

**Published:** 2020-05-22

**Authors:** Marcela Araujo Castro, Bruna Piloto, Caio Julio Cesar dos Santos Fernandes, Carlos Jardim, William Salibe Filho, Francisca Gavilanes Oleas, Jose Leonidas Alves, Luciana Tamie Kato Morinaga, Susana Hoette, Mario Terra Filho, Orival Freitas Filho, Fabio Biscegli Jatene, Rogerio Souza

**Affiliations:** 1 Pulmonary Division, Heart Institute, Hospital das Clinicas da Faculdade de Medicina da Universidade de Sao Paulo, Sao Paulo, Brazil; 2 Cardiovascular Surgery Division, Heart Institute, Hospital das Clinicas da Faculdade de Medicina da Universidade de São Paulo, São Paulo, Brazil; Universita degli Studi di Bologna, ITALY

## Abstract

Chronic thromboembolic pulmonary hypertension (CTEPH) is a rare complication of acute pulmonary embolism, characterized by non-resolving fibro-thrombotic obstructions of large pulmonary arteries. Pulmonary endarterectomy (PEA) is the treatment of choice for the disease, significantly improving survival. Patients with worse hemodynamic profile have worse prognosis after surgery, raising the question of whether the use of medical therapy prior to surgery to optimize hemodynamics could improve outcomes. The aim of this study was to evaluate the role of medical therapy pre-PEA, according to the hemodynamic profile at the diagnosis.

We retrospectively analyzed all patients submitted to PEA, from January 2013 to December 2017. Functional, clinical and hemodynamic data were collected to evaluate the main prognostic determinants. Patients were stratified according to the hemodynamic severity and use of targeted therapies prior to surgery.

A total of 108 patients were included. Thirty-five patients (32,4%) used targeted therapy pre-PEA. The use of medical therapy delayed the surgical procedure by about 7 months. There was no difference in overall survival between patients that received targeted therapy and those treated only with supportive therapy (87.8% vs 80.3%, respectively, p = 0.426). Nevertheless, when analyzing the group of patients with severe hemodynamic impairment, defined by low cardiac output(<3.7L/min) at baseline, patients treated with targeted therapies presented a significantly better one-year survival.

In higher-risk CTEPH patients, characterized by the presence of low cardiac output, the use of targeted therapies prior to PEA was associated with better outcome, suggesting a potential role for pre-operative use of medical treatment in this particular subgroup.

## Introduction

Chronic thromboembolic pulmonary hypertension (CTEPH) is a rare complication of acute pulmonary embolism (PE). It is caused by non-resolving fibro-thrombotic obstructions of large pulmonary arteries, combined with small vessel arteriopathy [1]. Both proximal and small vessel obstruction increase pulmonary vascular resistance (PVR), leading to progressive pulmonary hypertension, right heart failure and ultimately death [2].

Pulmonary endarterectomy (PEA) is the treatment of choice for the disease. Nevertheless, operability is determined by multiple factors from hemodynamic profile to the expertise of the surgical team and available resources [3]. Even considering that a significant proportion of patients have persistent pulmonary hypertension after surgery [4], the survival of non-operated patients is significantly worse. Delcroix et al. demonstrated that non-operated patients’ survival at 3 years was only 70% *vs* 89% in operated patients, despite having similar severity regarding functional class and hemodynamics at diagnosis [2].

There is an appealing rationale for the use of medical therapy before surgery as a way to optimize the hemodynamic condition, thus potentially improving the morbimortality associated with the procedure. However, such approach was previously tested in a large retrospective study; the use of pulmonary arterial hypertension (PAH) targeted therapies was associated with a significant delay in referral for definitive surgical treatment with no benefit in the outcomes [4]. More recently, similar results were described in a prospective international registry of CTEPH [2], reinforcing the lack of evidence supporting the routine use of medical therapy pre-PEA.

Nonetheless, a question that still remains is whether patients with more severe hemodynamic impairment could benefit from such strategy of attempting to improve hemodynamics prior to the surgical treatment [5].

The aim of this study was to evaluate the role of medical therapy in CTEPH patients pre-PEA according to the hemodynamic profile at the diagnosis.

## Methods

### Study design

All patients submitted to PEA, from January 2013 to December 2017, were included in this retrospective study. Baseline clinical, functional, and hemodynamic data were collected. Patients were stratified according to the use of PAH targeted therapies pre-PEA and also according to the baseline hemodynamic profile.

The diagnosis of CTEPH was established according to the current guidelines [6], based on the presence of multiple perfusion defects at ventilation/perfusion pulmonary scintigraphy, confirmed by the use of computed tomography (CT) scan and pulmonary angiography, in the presence of mean pulmonary artery pressure ≥ 25 mmHg with normal pulmonary artery occlusion pressure. At diagnosis, patients had at least 3 months of effective anticoagulation therapy.

The surgical procedures were performed through median sternotomy to allow bilateral endarterectomy. Cardiopulmonary bypass was initiated after cannulation of the ascending aorta and superior and inferior vena cava and progressive cooling up to 15°C with neuroprotection. Circulatory arrest was carried out in periods limited to 20 minutes at a time, with intervals of reperfusion. At the end of the bilateral endarterectomy, circulation with rewarming was resumed.

The study was approved by the Institutional Review Board of the University of Sao Paulo Medical School Hospital (Hospital das Clinicas da Faculdade de Medicina da Universidade de Sao Paulo) (protocol number 85547515.3.0000.0068) and all data have been fully anonymized before analysis.

### Statistical analysis

Data analysis was performed using the SPSS 21 statistical package (SPSS, Inc). All continuous variables are expressed as mean ± SD and compared using Student t test. Categorical data are presented as proportions and compared using chi-square or Fisher exact test, as appropriate.

All-cause mortality was used because of the lack of information about the specific cause of death in several cases. No patients were lost to follow-up during the study period. One-year survival was estimated using the Kaplan-Meier method. Patients were stratified according to the use of targeted therapies pre-PEA and also according to the median cardiac output. The log-rank test was used for curve comparison. Univariate analysis based on the proportional hazards model was used to examine the relationship between survival and selected baseline demographic, functional, and hemodynamic variables. Results are expressed as hazard ratios with 95% confidence intervals. Multivariate analysis was used to examine the independent effect on survival of variables select at the univariate analysis. A P value < 0.05 was considered statistically significant.

## Results

### Study population

A total of 108 consecutive patients who underwent to PEA surgery were enrolled in this retrospective study. Thirty-five patients (32,4%) had used targeted therapy pre-PEA.

Baseline characteristics, according to the use of targeted therapy pre-PEA are showed in Table 1.

**Table 1:** Baseline characteristics according to the use of targeted therapies.

	Total (n = 108)	Pre-PEA targeted therapy (n = 35)	Pre-PEA supportive therapy (n = 73)	P Value
Male gender	37 (34.3%)	9 (25.7%)	28 (38.4%)	0.076
Age (years)	46.2 ± 15.2	44.1 ± 14.1	47.1 ± 15.7	0.333
NYHA Functional Class				0.020
I	6 (5.9%)	3 (9.2%)	3 (4.4%)	
II	41 (40.2%)	8 (24.2%)	33 (47.8%)	
III	34 (33.3%)	11 (33.3%)	23 (33.3%)	
IV	21 (20.6%)	11 (33.3%)	10 (14.5%)	
Hemodynamics				
RAP (mmHg)	13.0 ± 6.2	13.3 ± 6.4	12.8 ± 6.8	0.679
mPAP (mmHg)	50.8 ± 13.0	54.3 ± 13.7	49.0 ± 12.4	0.048
PAOP (mmHg)	14.6 ± 5.9	14.7 ± 6.1	14.6 ± 5.9	0.916
CO (L/min)	3.9 ± 1.3	3.4 ± 0.9	4.1 ± 1.4	0.003
PVR (WU)	10.5 ± 5.5	12.5 ± 6.2	9.4 ± 4.7	0.110
BNP (pg/dL)	268.5 ± 283.2	284.3 ± 211.9	260.5 ± 314.4	0.687
6MWD (m)	365.9 ± 109.6	352.8 ± 106.8	373.0 ± 111.8	0.492

Patients who received targeted therapy had a more severe profile with higher mean pulmonary artery pressure (mPAP), lower cardiac output (CO) and worse functional class.

In the group of treated patients, 17 (48,6%) received a phosphodiesterase-5 inhibitor (PDE5i), 4 patients (11,4%) received an endothelin receptor antagonist (ERA) and 14 patients (40%) received upfront combination therapy with PDE5i + ERA. The use of medical therapy delayed the surgical procedure for about 7 months.

### Outcome

**Fig 1:**
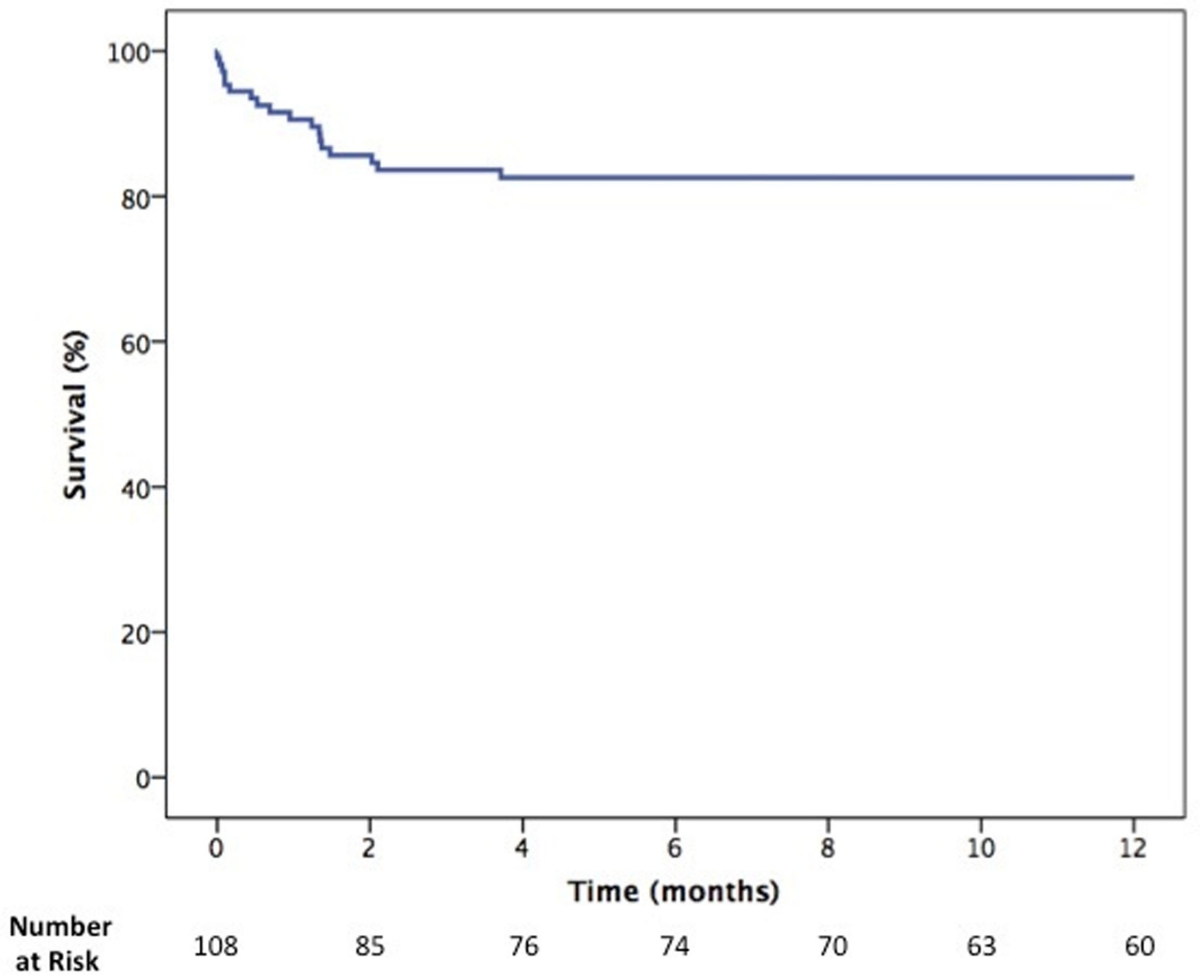
Overall 1-year survival.

**Fig 2:**
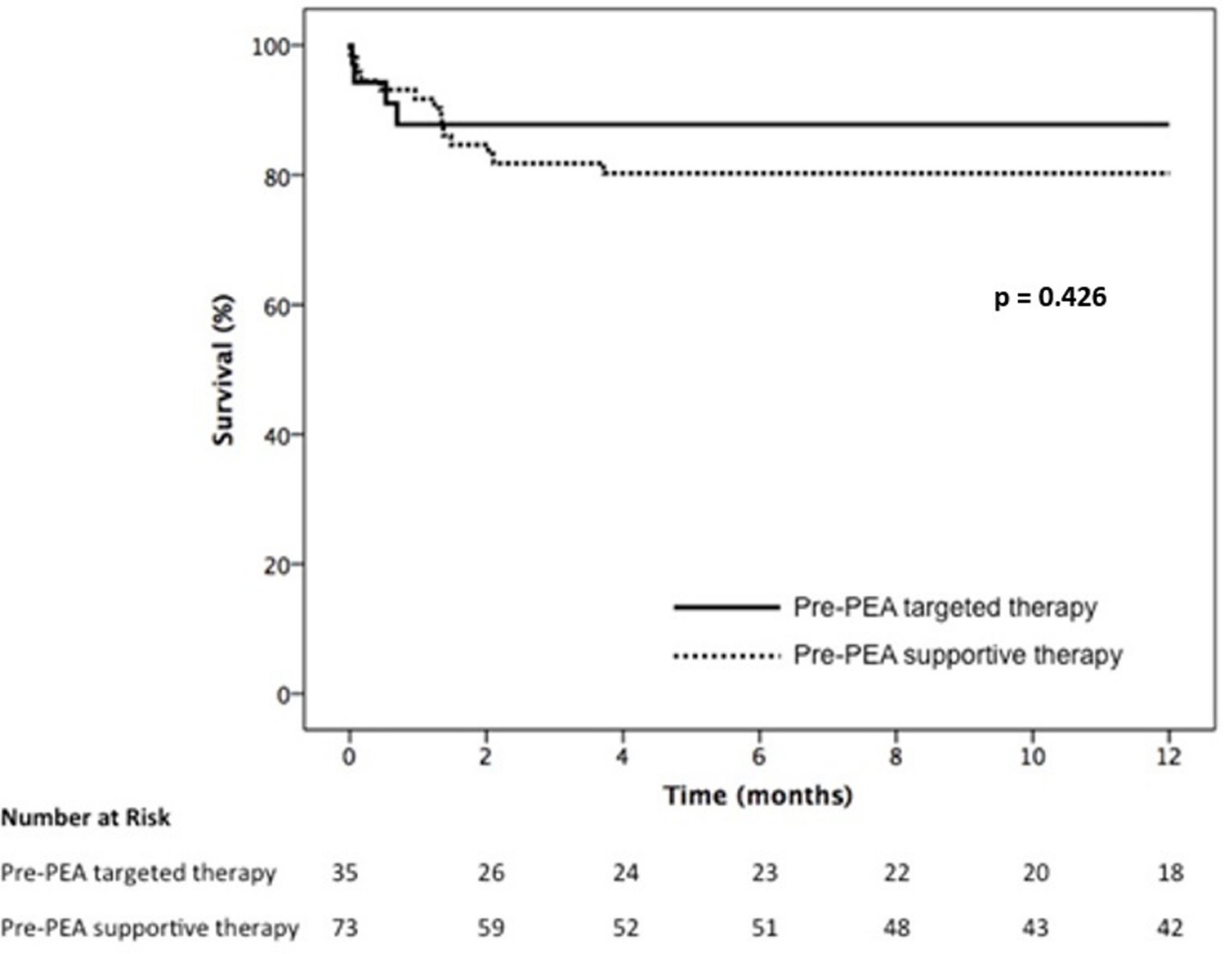
One-year survival according to the use of pre-PEA therapy.

One-year survival curves of the whole cohort and according to the use of targeted therapy are presented in Figs 1 and 2, respectively. The overall 1-year survival was 82.5%

There was no difference in survival between patients who received targeted therapy and those treated only with supportive therapy with anticoagulants and diuretics (87.8% vs 80.3%, respectively, p = 0.426). Most deaths occurred in the first two months after surgery, having infection associated with sustained right ventricular dysfunction as the major cause of death (71.6% of all cases).

Univariate analysis demonstrated that older age, higher BNP and lower CO were significantly associated with increased mortality (Table 2).

**Table 2:** Univariate analysis of selected baseline variables.

	Hazard Ratio (95% CI)	P Value
Male gender	1.292 (0.501–3.334)	0.596
Age (years)	1.045 (1.011–1.079)	0.008
NYHA functional class		
II	0.468 (0.052–4.187)	0.497
III	0.903 (0.109–7.499)	0.924
IV	1.554 (0.187–12.919)	0.683
Hemodynamics		
mPAP (mmHg)	0.995 (0.957–1.033)	0.779
CO (L/min)	0.393 (0.195–0.794)	0.009
PVR (WU)	1.053 (0.959–1.155)	0.280
BNP (pg/dL)	1.001 (1.000–1.003)	0.026
6MWD (m)	0.997 (0.992–1.003)	0.377
Pre-PTE targeted therapy	0.640 (0.210–1.943)	0.431

The multivariate analysis including these variables demonstrated that CO was independently associated with prognosis (Table 3).

**Table 3:** Multivariate analysis of selected baseline variables.

	Hazard Ratio (95% CI)	P Value
Age (years)	1.053 (0.996–1.112)	0.070
CO (L/min)	0.374 (0.158–0.884)	0.025
BNP (pg/dL)	1.001 (0.999–1.003)	0.285

**Fig 3:**
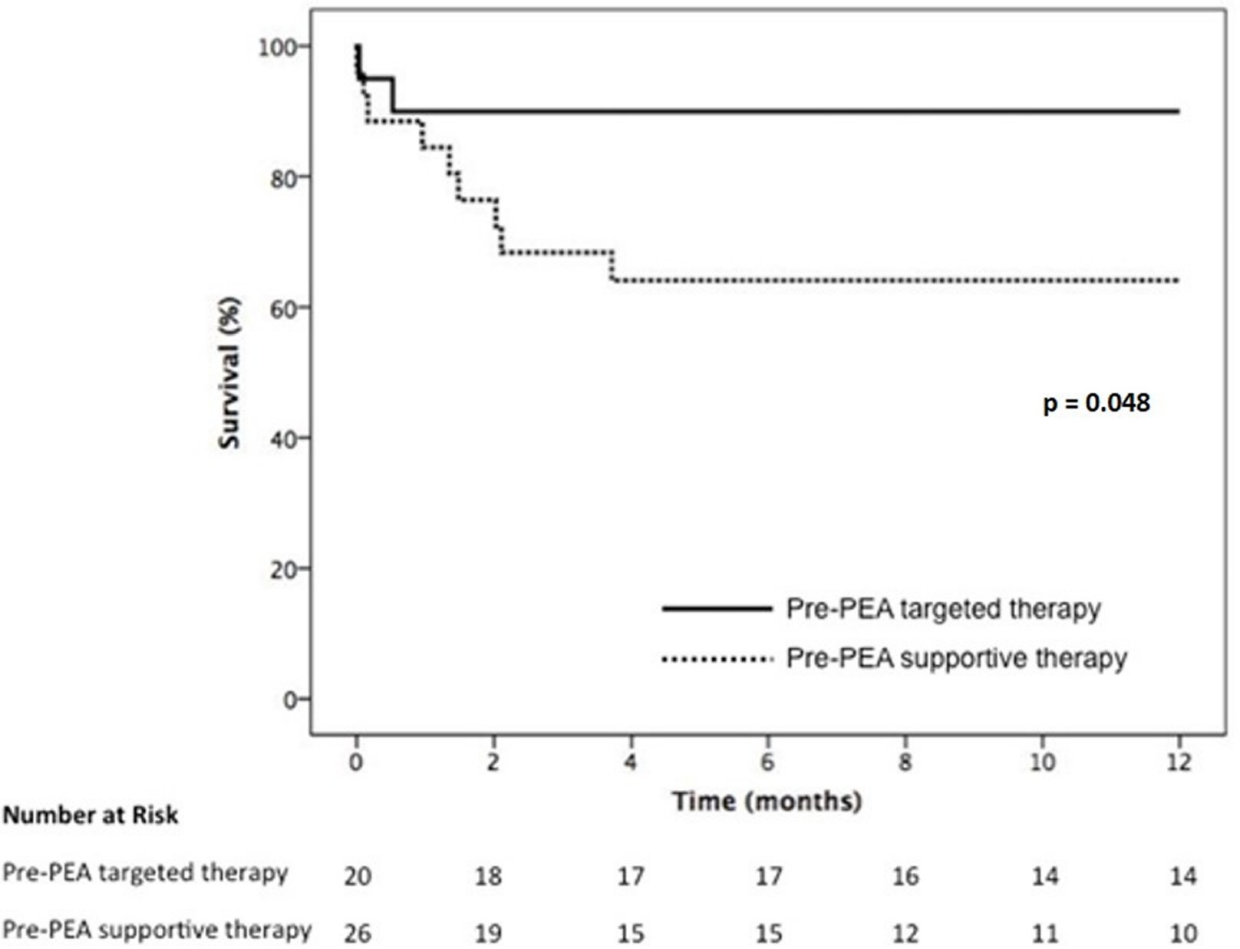
One-year survival according to the use of pre-PEA therapy in patients with low cardiac output (< 3.75 L ∕ min).

When analyzing only the group of patients with CO below the median value (3.75 L/min), thus with a more severe hemodynamic impairment at baseline, treated and non treated patients had similar baseline profile (Table 4) and the same postoperative management but the use of targeted therapy was associated with a significantly better one-year survival (89.1% vs 64.1%) (Fig 3).

**Table 4:** Baseline characteristics of patients with low cardiac output according to the use of targeted therapies.

	Pre-PEA targeted therapy (n = 20)	Pre-PEA supportive therapy (n = 26)	P Value
Age (years)	44.8 ± 13.0	50.8 ± 13.2	0.12
Hemodynamics			
RAP (mmHg)	13.5 ± 5.9	13.0 ± 6.9	0.79
mPAP (mmHg)	55.4 ± 11.8	49.4 ± 11.4	0.88
PAOP (mmHg)	14.7 ± 6.5	14.6 ± 7.6	0.98
CO (L/min)	2.82 ± 0.5	2.94 ± 0.5	0.42
PVR (WU)	14.5 ± 4.9	12.7 ± 4.6	0.22
BNP (pg/dL)	339.2 ± 191.4	442.4 ± 387.8	0.28
6MWD (m)	340.0 ± 90.7	333.2 ± 134.3	0.88

## Discussion

Our study showed that in patients with severe hemodynamic impairment, the use of targeted therapies prior to PEA was significantly associated with better outcome. Nevertheless, as previously demonstrated, this benefit was not evident if the whole population of CTEPH patients was considered, suggesting that the use of medical therapy pre-PEA could be an option, but only for well-selected patients.

Our patient population presented similar hemodynamic severity to that included in previous studies reporting the use of medical therapy before PEA. Similarly, the group treated with targeted therapies had worse functional capacity and hemodynamic profile compared to the group receiving supportive therapy pre-PEA [2, 4].

The rationale for using medical therapy in CTEPH has evolved in the last decade. It is now clear that patients with persistent or residual pulmonary hypertension after PEA, inoperable patients or patients with an unacceptable surgical risk–benefit ratio might benefit from the use of specific medical therapy [3]. Riociguat, a soluble guanylate cyclase stimulator, is approved for this setting based on the results of the Chronic Thromboembolic Pulmonary Hypertension Soluble Guanylate Cyclase–Stimulator Trial 1 (CHEST-1) [7]. More recently, the results of the macitentan for the treatment of inoperable chronic thromboembolic pulmonary hypertension (MERIT-1) trial further reinforced that in CTEPH patients considered to be inoperable, medical treatment resulted in improved hemodynamics and functional capacity[8]. Other uncontrolled case series with different compounds showed similar results [9–11]. Furthermore, the presence of microvasculopathy in CTEPH is becoming more evident [12]. Several forms of pulmonary vascular lesions are well-known in the setting of CTEPH [13, 14]. More recently, Dorfmuller et al. demonstrated that even obstructed territories presented important microvascular remodeling; moreover, the authors also described significant anastomoses between the systemic and pulmonary circulation that could be related to the pathophysiology of this microvasculopathy [1].

Nevertheless, although these aspects support the use of medical therapy in CTEPH, the largest series evaluating the use of targeted therapies prior to PEA failed to demonstrate any benefit on outcomes [2, 4]. Additionally, the attempt to use medical therapy was significantly associated with a delay in referral for surgery [4]. This delay is explained by the usual time to evaluate treatment efficacy of 12 to 16 weeks. Similarly, in our study, surgery was delayed for about 7 months when medical treatment was used and no benefit on survival was observed when the whole cohort was considered. Altogether, these results support the current guidelines, limiting the use of medical therapy for non-surgical patients or those with residual pulmonary hypertension after the procedure [6].

However, none of these studies stratified patients according to the severity of hemodynamic impairment [5]. It is well recognized that the hemodynamic profile prior to surgery is associated with outcomes. Data form the international CTEPH registry demonstrated that patients with pulmonary vascular resistance (PVR) higher than 1200 dyn·s·cm^–5^ had a significantly worse survival than patients with better preserved hemodynamic pattern [15]. Jensen et al. evidenced that a PVR > 1000 dyn·s·cm^–5^ was associated with increased morbidity and mortality [4]. These data at least raise the question of whether higher-risk patients could benefit from pre-operative treatment. The multivariate analysis in our cohort evidenced that cardiac output was the best predictor of survival; therefore, we decided to evaluate the patient population with low cardiac output as the higher-risk group. In this subgroup of patients, the use of medical therapy aiming to optimize hemodynamics prior to PEA resulted in a significantly better outcome, suggesting than in higher-risk groups, pre-operative use of targeted therapy might have a beneficial role.

Our study has limitations that must be acknowledged. In our single-center study, the decision to pre-operatively treat was made at the attending physician discretion and not based on an established protocol; nevertheless, it is clear that this decision was mostly based on a higher severity of the disease. Patients were then treated with off-label PAH-targeted therapies (sildenafil, bosentan or ambrisentan), according to drug availability in the country, instead of receiving riociguat, the only compound approved for use in CTEPH, although not as pre-operative treatment. No specific protocol for choosing the upfront treatment strategy (monotherapy or combination therapy) was applied, preventing any recommendation in this sense. Still, our treated group presented significant benefit despite the worse hemodynamic profile at baseline.

## Conclusions

In conclusion, in higher-risk patients characterized by the presence of low cardiac output, the use of targeted therapies prior to PEA was associated with better outcome, suggesting a potential role for pre-operative use of medical treatment in this group. Nevertheless, our results reinforce that except for this higher risk group, the use of medical therapy prior to PEA is associated with delayed referral for surgery without influencing outcomes.

## Supporting information

S1 Data (XLSX)
